# Multiple Chronic Medical Conditions and Health-Related Quality of Life in Older Adults, 2004–2006

**DOI:** 10.5888/pcd10.120282

**Published:** 2013-09-26

**Authors:** John P. Barile, William W. Thompson, Matthew M. Zack, Gloria L. Krahn, Willi Horner-Johnson, Sonya E. Bowen

**Affiliations:** Author Affiliations: William W. Thompson, Matthew M. Zack, Gloria L. Krahn, Centers for Disease Control and Prevention, Atlanta, Georgia; Willi Horner-Johnson, Oregon Health and Science University, Portland, Oregon; Sonya E. Bowen, Centers for Medicare and Medicaid Services, Baltimore, Maryland.

## Abstract

**Introduction:**

Understanding longitudinal relationships among multiple chronic conditions, limitations in activities of daily living, and health-related quality of life is important for identifying potential opportunities for health promotion and disease prevention among older adults.

**Methods:**

This study assessed longitudinal associations between multiple chronic conditions and limitations in activities of daily living on health-related quality of life among older adults (≥65 years) from 2004 through 2006, using data from the Medicare Health Outcomes Survey (N = 27,334).

**Results:**

Using a longitudinal path model, we found the numbers of chronic conditions at baseline and 2-year follow-up were independently associated with more limitations in activities of daily living at 2-year follow-up. In addition, more limitations in activities of daily living at 2-year follow-up were associated with worse health-related quality of life during the follow-up time period. The association between multiple chronic conditions and indices of health-related quality of life was mediated by changes in limitations in activities of daily living.

**Conclusion:**

Both baseline and new multiple chronic conditions led to worse health in terms of activities of daily living and health-related quality of life and should be considered important outcomes to intervene on for improved long-term health. In addition, public health practitioners should consider addressing classes of multiple chronic conditions by using interventions designed to reduce the emergence of multiple chronic conditions, such as physical activity, reductions in smoking rates, and improved and coordinated access to health care services.

## Introduction

Health-related quality of life (HRQOL) is an important health outcome and is typically assessed by using self-reported physical and mental health indices (1–3). In addition, the US Department of Health and Human Services (HHS) has prioritized measuring HRQOL during the next decade to monitor goals and objectives designed to improve population health in the United States (4).

HHS has developed a research agenda to investigate relationships between multiple chronic conditions (MCCs) and other health outcomes (5,6). HHS defines MCCs as the presence of 2 or more chronic conditions, which may include conditions such as hypertension, arthritis, diabetes, asthma, and depression. The prevalence of MCCs is high among older adults; 62% of adults over 65 years of age report having at least 2 chronic conditions (7). HHS has outlined the need for additional longitudinal epidemiologic studies to determine the effect of MCCs on health outcomes. Of particular interest to HHS is the association between MCCs and HRQOL (5).

Functional limitations are associated with MCCs, especially those most prevalent in older adults (eg, arthritis, diabetes, cardiovascular disease) and HRQOL (8–13). Functional limitations are often measured by activities of daily living (ADLs) (14) and have been associated longitudinally with home care visits and hospitalizations (8,9). Furthermore, older adults with at least 1 ADL limitation during baseline assessments were nearly twice as likely as individuals with no ADL limitations to report greater dependence on other individuals at follow-up (8).

The relationships between MCCs, functional limitations, and HRQOL include complex relationships that likely change over time and are reciprocal in nature (12,13). MCCs are associated with worse HRQOL, and the impact of MCCs may depend on whether an individual has ADL limitations (13). A cross-sectional study found that older adults with no ADL limitations may have the most to gain by preventing new chronic conditions, which in turn appear to prevent new ADL limitations (13). Understanding the progression from development of MCCs and ADL limitations to subsequently poorer HRQOL can help health professionals develop prevention and intervention strategies to interrupt this cycle. Our investigation extends previous work by testing a longitudinal model that seeks to assess how MCCs affect older adults’ ability to complete their ADLs and the potential impact of ADL limitations on HRQOL over a 2-year period.

This study uses data from the Medicare Health Outcomes Survey (HOS) among older adults to test the following 3 hypotheses:

1. The number of MCCs at baseline is associated with more ADL limitations at follow-up.
2. ADL limitations at follow-up are associated with changes in HRQOL.
3. MCCs at baseline and at follow-up are both directly and indirectly associated with changes in HRQOL.

## Methods

### Data source

The Centers for Medicare and Medicaid Services (CMS) monitors longitudinal health outcomes including HRQOL through Medicare Advantage (MA) Plans (www.Medicare.gov). These plans include both seniors 65 years or older and younger people with disabilities enrolled in Medicare Part A and Part B who have chosen to enroll in managed care rather than fee-for-service coverage of hospital and medical care. Our study used data from the CMS-administered Medicare Health Outcomes Survey (HOS), which is used to inform quality improvement programs and to assess performance in Medicare managed care.

HOS involves mailing questionnaires to randomly selected MA Plan enrollees each year; nonrespondents receive a computer-assisted telephone follow-up interview. Baseline respondents receive follow-up surveys 2 years later. For MA Plans with an enrolled population of more than 1,000, a simple random sample of 1,000 members receives the HOS, and for MA Plans with fewer than 1,000 members, all members receive the HOS. Excluded from the HOS are those not continuously enrolled in the same plan for at least 6 months and those with end-stage renal disease. This process resulted in a sampling of 155 health care organizations.

### Participants

Study subjects came from the HOS Cohort 7 who completed the baseline survey in 2004 (N = 99,649 of 159,311 surveys; 65.3% response rate) and the follow-up survey in 2006 (N = 57,214 of 69,865; 83.0% response rate). Please see http://www.hosonline.org/Content/SurveyResults.aspx for more information on the sampling method. We excluded from the analyses respondents younger than 65 years old because they all were receiving disability benefits and were likely to differ from older respondents by background characteristics (n = 10,888). We also excluded any respondents who used a proxy to complete the survey at either baseline or follow-up because proxy responses differ from those collected directly from patients with similar medical histories (n = 26,569) and those with missing data (n = 12,664) on any of the variables under study (15). This process left a sample of 27,334 participants with complete baseline and follow-up data. We limited our analytical sample to those with complete data and did not attempt to impute missing data because data were not missing at random (eg, 1,084 participants disenrolled, 529 died before follow-up). Consequently, these findings can only be generalized to healthy populations of older adults because those with missing data or with proxy information reported significantly more physically (*P* < .001) and mentally (*P* < .001) unhealthy days.

The final sample of older adults were found to be demographically similar to 2004 national census figures for adults older than 65 years of age (16). The final sample was composed of a white majority (92%) that was 57% female. Fifty-eight percent were aged 65 to 74 years, 58% were high school graduates or less (19% had a college degree), and 63% had an annual income of less than $30,000 (13% reported incomes of at least $50,000).

### Measures

**Multiple chronic conditions**

The HOS asked whether participants had ever been diagnosed with a range of chronic medical conditions. At baseline, each condition was coded as 0 (never) or 1 (diagnosed) and a summary score was created that represented the sum of the number of medical conditions experienced by each participant. Using the follow-up data, we created a dichotomous (1 = yes, 0 = no) variable that represented whether a person reported at least 1 additional chronic condition that was not reported at baseline.

The analyses also controlled for numerous potential confounding medical conditions and behaviors including chronic low back pain, cancer treatment, and smoking status. The measure of chronic low back pain asked: “In the past 4 weeks, how often has low back pain interfered with your usual daily activities (work, school or housework)?” Responses for this question on a Likert-type scale ranged from 1 (none of the time) to 5 (all of the time). Cancer treatment was assessed by asking participants if they were currently under treatment (no = 0, yes = 1) for colon, lung, breast, or prostate cancer. Smoking status was assessed by asking participants if they currently smoke every day, some days, or not at all.

**Activities of daily living**

A 6-item ADL limitations measure was used to assess study participants’ level of functioning at baseline and follow-up. The 6 items share a common stem, followed by a list of specific activities: “Because of a health or physical problem, do you have any difficulty doing the following activities [bathing, dressing, eating, getting in or out of chairs, walking, or using the toilet]?” (14). Respondents selected 1 (I am unable to do this activity), 2 (yes, I have difficulty), or 3 (no, I do not have difficulty). These items were rescored so that 0 was no difficulty, 1 was yes, I have difficulty, and 2 was I am unable to do this activity; they were then summed to form a total score ranging from 0 to 12 (Cronbach’s α = .81 at baseline; α = .80 at follow-up).

The Centers for Disease Control and Prevention (CDC) has developed 4 core HRQOL measures that have undergone cognitive testing and have demonstrated content validity, construct validity, criterion validity with the Short Form-36, predictive validity, test-retest reliability, and internal consistency (2,17). Two of the 4 core HRQOL measures were used as outcomes in this study. The first question, physically unhealthy days, asks, “Now thinking about your physical health, which includes physical illness and injuries, for how many days during the past 30 days was your physical health not good?” The second question, mentally unhealthy days, asks, “Now thinking about your mental health, which includes stress, depression, and problems with emotions, for how many days during the past 30 days was your mental health not good?” These items were chosen because they have been shown to be less biased for assessing HRQOL among people with functional limitations than other, similar measures of HRQOL (18,19).

**Demographics**

The following demographic data from survey respondents were included as potential confounders in all analyses: age, annual household income, educational attainment, race, and sex. Participants’ birthdates were used to calculate an exact age at time of assessment. Participant self-reported annual household income was an ordinal variable ranging from 1 (less than $5,000) to 9 ($100,000 or more).

### Data analyses

All hypotheses were tested using the path model (Figure). Analyses were conducted by using a structural equation modeling framework with Mplus v6.0 statistical software (Muthén and Muthén, Los Angeles, California). We used a full information, robust maximum likelihood estimator (*mlr*) to obtain parameter estimates and standard errors robust to nonnormality. A single model was used to simultaneously test hypothesis 1: the associations between initial MCCs and ADL limitations at follow-up (while controlling for baseline ADLs); hypothesis 2: the association between ADL limitations at follow-up and changes in HRQOL (by controlling for baseline HRQOL); and hypothesis 3: direct and indirect associations between initial and follow-up MCCs and changes in HRQOL over a 2-year period. The variances associated with both physically and mentally unhealthy days were estimated by using negative binomial regression that allows for the independent specification of the mean and the variance (20) because the variances of the unhealthy days outcomes exceeded their means. Indirect effects of all predictor variables on the outcomes via mediators were estimated by using Monte Carlo methods for assessing mediation with R statistical software, R v2.11 (StataCorp LP, College Station, Texas), using tools developed by Selig and Preacher (21). All analyses controlled for age, education, race/ethnicity, sex, smoking status, cancer treatment status, and back pain.

**Figure:**
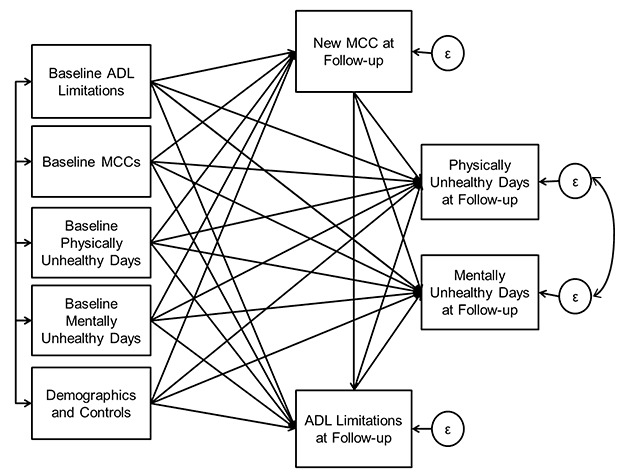
Estimated mediation model at baseline and at follow-up for multiple chronic conditions (MCC), limitations in activities of daily living (ADLs), and unhealthy days. Estimated error variance is indicated by ε.

## Results

We calculated descriptive statistics for each of the conditions under study as well as all outcome variables (Table 1). Individuals in our sample reported back pain that ranged between none and all of the time in the past 4 weeks (mean = 1.81, SD, 1.08). Individuals in the sample also reported cancer treatment for colon (1%), lung (<1%), breast (2%), and/or prostate (3%) cancer. The sample included older adults that reported either smoking every day (7%), some days (3%), or not at all (90%).

**Table 1:** Descriptive Statistics for Selected Medical Conditions, Limitations in Activities of Daily Living, and Health-Related Quality of Life (N = 27,334)^a^

Characteristic	Baseline	Follow-up
Ever been diagnosed		
Stroke	1,523 (6)	1,779 (7)
Respiratory condition	3,272 (12)	3,568 (13)
Stomach or intestinal condition	1,181 (4)	1,166 (4)
Arthritis (hand, wrist, hip, or knee)	14,231 (52)	14,171 (52)
Diabetes mellitus	4,646 (17)	5,342 (20)
Any cancer	3,964 (15)	4,519 (17)
Angina or coronary artery disease	3,775 (14)	3,834 (14)
Congestive heart failure	1,501 (6)	1,890 (7)
Heart attack	2,474 (9)	2,713 (10)
Other heart condition	5,334 (20)	5,759 (21)
Sciatica	5,890 (22)	5,373 (20)
**Total number of conditions**		
0	5,808 (21)	5,523 (20)
1	8,178 (30)	7,860 (29)
2	6,363 (23)	6,412 (24)
3	3,654 (13)	3,805 (14)
4	1,790 (7)	1,944 (7)
5	860 (3)	979 (4)
6	412 (2)	485 (2)
7 or more conditions	269 (1)	326 (1)
Additional multiple chronic condition at follow-up	NA	9,977 (37)
**No. of ADL limitations^b^**		
0 Limitations	18,353 (67)	19,202 (70)
1 Limitation	3,866 (14)	3,257 (12)
2 Limitations	2,878 (11)	2,439 (9)
3 Limitations	933 (3)	1,029 (4)
4 Limitations	630 (2)	600 (2)
5 Limitations	348 (1)	416 (2)
6 or More limitations	326 (1)	391 (1)
**No. of chronic conditions (range, 0–11), mean (SD)**	1.8 (1.5)	1.8 (1.4)
**ADL limitations (range, 0–12), mean (SD)**	0.7 (1.4)	0.7 (1.4)
**No. of unhealthy days (range, 0–30), mean (SD)**		
Physical health	4.0 (8.4)	4.6 (8.6)
Mental health	1.9 (5.6)	2.3 (5.9)

Source: Data are from the Centers for Medicare and Medicaid Services–administered Medicare Health Outcomes Survey, 2004–2006.

Abbreviation: NA, not applicable; ADL, activities of daily living.

a Data presented are n (%) unless otherwise specified.

b Limitations in activities of daily living were scored on a 0–12 scale. For each of the 6 activities, respondents received a score of 0 if they reported no difficulty with the activity, 1 for reporting some difficulty, and 2 if they were unable to do this activity; these scores were then summed.

Our model resulted in significant associations along the paths representing our 3 hypotheses (Table 2). In support of hypothesis 1, more MCCs at baseline were associated with more ADL limitations at follow-up (*b* = 0.09, *P* < .001), and additional chronic conditions at follow-up were associated with more ADL limitations at follow-up (*b* = 0.21, *P* < .001). For hypothesis 2, ADL limitations at follow-up were associated with more physically (*b* = 0.40, *P* < .001) and mentally (*b* = 0.30, *P* < .001) unhealthy days at follow-up, after controlling for baseline levels of unhealthy days (Table 3). In support of hypothesis 3, MCCs at baseline were directly associated with more physically (*b* = 0.14, *P* < .001) and mentally (*b* = 0.08, *P* < .001) unhealthy days at follow-up. Baseline MCCs significantly affected changes in physical (95% CI, 0.036–0.048) and mental health (95% CI, 0.021–0.033) indirectly via ADLs at follow-up. Follow-up MCCs also significantly affected changes in physical (95% CI, 0.175–0.226) and mental health (95% CI, 0.075–0.123) indirectly via ADLs at follow-up.

**Table 2:** Longitudinal Associations Between Predictors of ADL Limitations and the Reporting of at Least 1 New Chronic Condition at Follow-up (N = 27,334)

Demographic and Health Status	Reported at Least 1 New Condition at Follow-up			ADL Limitations at Follow-up	
	*b* (SE)	*P*	OR^a^	*b* (SE)	*P*
**Age**	0.01 (0)	<.001	1.01	0.02 (0)	<.001
Income (on a 1–9 scale^b^)	−0.01 (0.01)	.11	0.99	−0.03 (0)	<.001
**Education**					
Less than high school diploma	0.04 (0.04)	.24	1.04	−0.03 (0.02)	.17
High school diploma or GED	Reference			Reference	
Some college	−0.03 (0.03)	.42	0.97	0.02 (0.02)	.28
College degree	−0.09 (0.04)	.02	0.91	0.03 (0.02)	.18
**Race**					
White	Reference			Reference	
Black	−0.05 (0.06)	.42	0.95	0.08 (0.04)	.03
Other race	−0.13 (0.07)	.08	0.88	−0.04 (0.03)	.31
**Sex**					
Men	Reference			Reference	
Women	−0.20 (0.03)	<.001	0.82	−0.01 (0.02)	.55
**Smoking status**					
Nonsmoker	Reference			Reference	
Smoke every day	0.15 (0.05)	<.001	1.16	0.05 (0.03)	.10
Smoke sometimes	−0.05 (0.08)	.48	0.95	0.07 (0.04)	.12
**Other health measures**					
Colon cancer treatment	−0.02 (0.14)	.91	0.98	0.15 (0.11)	.18
Lung cancer treatment	0.30 (0.23)	.20	1.35	−0.12 (0.17)	.49
Breast cancer treatment	−0.09 (0.10)	.36	0.91	−0.03 (0.06)	.60
Prostate cancer treatment	0.05 (0.08)	.53	1.05	−0.05 (0.05)	.28
Back pain (on a scale of 1−5^c^)	0.08 (0.01)	<.001	1.08	0.13 (0.01)	<.001
**Health-related quality of life, ADL, and MCCs at baseline and follow-up**					
Baseline physically unhealthy days	0.01 (0)	<.001	1.01	0.02 (0)	<.001
Baseline mentally unhealthy days	0.01 (0)	<.001	1.01	0.01 (0.00)	.001
Baseline MCCs (range, 0−11^d^)	0.01 (0.01)	.44	1.01	0.09 (0.01)	<.001
Baseline ADL limitations (range, 0–12^e^)	0.03 (0.01)	.01	1.03	0.37 (0.02)	<.001
Follow-up new MCCs (0/1)^f^	NA			0.21 (0.02)	<.001

Source: Data are from the Centers for Medicare and Medicaid Services−administered Medicare Health Outcomes Survey, 2004–2006.

Abbreviations: ADL, activities of daily living; MCC, multiple chronic conditions; SE, standard error; OR, odds ratio; GED, general educational development diploma; NA, not applicable.

a ORs larger than 1 suggest a higher probability of reporting at least 1 new chronic condition or an ADL limitation at follow-up.

b Income scale is represented by 1 = Less than $5,000; 2 = $5,000–$9,999; 3 = $10,000–$19,999; 4 = $20,000–$29,999; 5 = $30,000–$39,999; 6 = $40,000–$49,999; 7 = $50,000–$79,999; 8 = $80,000–$99,999; 9 = $100,000 or more.

c Back pain is represented by a Likert-type scale, 1 = none of the time, 2 = a little of the time, 3 = some of the time, 4 = most of the time, 5 = all of the time.

d This score represents the total number of chronic conditions reported by each participant, from 0 through 11. Zero represents individuals with no chronic conditions at baseline and 11 represents individuals who reported having been diagnosed with all of the chronic conditions listed in the survey.

e Limitations in activities of daily living were scored on a 0–12 scale. For each of the 6 activities, respondents received a score of 0 if they reported no difficulty with the activity, 1 for reporting some difficulty, and 2 if they were unable to do this activity; these scores were then summed.

f Zero represents no new MCCs at follow-up, 1 represents reporting at least 1 new MCC at follow-up.

**Table 3:** Longitudinal Associations Between Predictors and Mediators of Physically and Mentally Unhealthy Days at Follow-up (N = 27,334)

Demographic and Health Status	Physically Unhealthy Days at Follow-up				Mentally Unhealthy Days at Follow-up			
	*b* (SE)	*P* Value	IRR^a^	Δ Days b	*b* (SE)	*P* Value	IRR^b^	Δ Days b
**Age**	0.01 (0.00)	.05	1.01	0.05	0.00 (0.00)	.58	1.00	0.00
**Income (on a 1–9 scale^c^)**	−0.03 (0.01)	.002	0.97	−0.14	−0.04 (0.01)	<.006	0.96	−0.09
**Education**								
Less than high school diploma	0.05 (0.04)	.22	1.05	0.23	0.03 (0.05)	.64	1.03	0.07
High school diploma or GED	Reference				Reference			
Some college	−0.05 (0.04)	.20	0.96	−0.18	−0.03 (0.05)	.49	0.97	−0.07
College degree	−0.04 (0.04)	.43	0.97	−0.14	0.05 (0.06)	.43	1.05	0.12
**Race**								
White	Reference				Reference			
Black	0.04 (0.06)	.51	1.04	0.18	−0.06 (0.09)	.48	0.94	−0.14
Other race	−0.04 (0.09)	.63	0.96	−0.18	−0.10 (0.11)	.37	0.91	−0.21
**Sex**								
Male	Reference				Reference			
Female	0.10 (0.03)	.001	1.11	0.51	0.31 (0.04)	<.001	1.36	0.84
**Smoking status**								
Nonsmoker	Reference				Reference			
Smoke every day	0.07 (0.06)	.21	1.07	0.32	0.23 (0.07)	.002	1.25	0.59
Smoke sometimes	−0.03 (0.08)	.75	0.98	−0.09	0.06 (0.10)	.59	1.06	0.14
**Other health measures**								
Colon cancer treatment	0.08 (0.15)	.61	1.08	0.37	−0.05 (0.20)	.81	0.95	−0.12
Lung cancer treatment	0.41 (0.18)	.02	1.50	2.31	0.23 (0.27)	.38	1.26	0.61
Breast cancer treatment	0.07 (0.11)	.52	1.07	0.32	−0.36 (0.15)	.02	0.70	−0.70
Prostate cancer treatment	−0.03 (0.09)	.73	0.97	−0.14	−0.06 (0.11)	.57	0.94	−0.14
Back pain (1–5^d^)	0.19 (0.01)	<.001	1.21	0.97	0.13 (0.02)	<.001	1.14	0.33
**Health-related quality of life, ADL, and MCCs at baseline and follow-up **								
Baseline physically unhealthy days	0.03 (0)	<.001	1.03	0.14	0.01 (0)	<.001	1.01	0.02
Baseline mentally unhealthy days	0.02 (0)	<.001	1.02	0.09	0.10 (0)	<.001	1.10	0.23
Baseline MCCs (range, 0–11^e^)	0.14 (0.01)	<.001	1.15	0.69	0.08 (0.01)	<.001	1.08	0.19
Baseline ADL limitations (range, 0–12)	−0.02 (0.01)	.15	0.99	−0.05	−0.01 (0.02)	.46	0.99	−0.02
Follow-up new MCCs (0/1^f^)	0.50 (0.03)	<.001	1.65	3.00	0.33 (0.04)	<.001	1.40	0.94
Follow-up ADL limitations (range, 0–12)	0.40 (0.01)	<.001	1.49	2.26	0.30 (0.01)	<.001	1.35	0.82

Source: Data are from the Centers for Medicare and Medicaid Services–administered Medicare Health Outcomes Survey, 2004–2006.

Abbreviations: ADL, activities of daily living; IRR, incident rate ratios; GED, general educational development diploma; MCCs, multiple chronic conditions.

a Incident rate ratios greater than 1 suggest a higher probability of reporting physically or mentally unhealthy days at follow-up.

b “Δ Days” represents the change in the number of physically or mentally unhealthy days associated with reporting a 1-unit increase in the predictor variable.

c Income scale is represented by 1 = Less than $5,000; 2 = $5,000–$9,999; 3 = $10,000–$19,999; 4 = $20,000–$29,999; 5 = $30,000–$39,999; 6 = $40,000–$49,999; 7 = $50,000–$79,999; 8 = $80,000–$99,999; 9 = $100,000 or more.

d Back pain is represented by a Likert-type scale, 1 = none of the time, 2 = a little of the time, 3 = some of the time, 4 = most of the time, 5 = all of the time.

e This score represents the total number of MCCs reported by each participant, from 0 through 11. Zero represents individuals with no MCCs at baseline and 11 represents individuals who reported having been diagnosed with all of the chronic conditions listed in the survey.

f Zero represents no new MCCs at follow-up, 1 represents reporting at least 1 new MCC at follow-up.

Having more MCCs at baseline and reporting 1 or more additional medical conditions at follow-up were associated with more ADL limitations at follow-up. More ADL limitations at follow-up were, in turn, associated with worse HRQOL during the 2-year period. These associations can also be translated into differences in the number of unhealthy days experienced by older adults. The incident rate ratios (IRRs) associated with physically and mentally unhealthy days can be used to estimate the change in the dependent variable associated with 1 or more unit changes in the independent variable. For example, reporting 3 additional MCCs at baseline was directly associated with 2.41 additional physically unhealthy days ([IRR^3 ^× mean for physically unhealthy days at follow-up] – mean for physically unhealthy days at follow-up) and 0.63 additional mentally unhealthy days during the measure’s 30-day referent period. Reporting 1 or more new chronic conditions at follow-up (controlling for baseline MCCs) was associated with 2.99 more physically unhealthy days and 0.94 additional mentally unhealthy days at follow-up. Finally, reporting 3 additional ADL limitations at follow-up was associated with 10.69 additional physically unhealthy days and 3.42 additional mentally unhealthy days.

## Discussion

Findings from this study support all 3 of our hypotheses: 1) The number of MCCs at baseline and follow-up was associated with increases in ADL limitations at follow-up, 2) more ADL limitations at follow-up were associated with worse HRQOL as operationalized with physically and mentally unhealthy days, and 3) the association between MCCs and HRQOL was mediated by changes in ADLs. These findings provide the public health community with information regarding complex relationships between disease, disability, and HRQOL in older adults that may assist them in developing intervention strategies. These relationships can be characterized in the following manner: MCCs, both previous and incident, affect both ADLs and HRQOL. Therefore, intervening on MCCs at any point in time will likely improve both current and subsequent ADLs and HRQOL. This conclusion suggests that reducing rates of MCCs would have substantial global health benefits at any time and that interventions that reduce the likelihood of MCCs will have significant downstream benefits. These results highlight the need for broad evidence-based public health and clinical interventions that decrease rates of MCCs (for example, increasing rates of physical activity, improving diet and access to quality health care, and decreasing rates of smoking).

Because MCCs at baseline and new MCCs during follow-up were strongly associated with subsequent increases in limitations in ADLs, prevention efforts to reduce older adults’ likelihood of developing new MCCs could forestall increases in ADL limitations. These findings support previous work (10) that suggested that MCCs likely precede the presentation of ADL limitations in older adults. Furthermore, because our study showed that increases in ADL limitations were associated subsequently with poorer HRQOL, the benefits of treatment and prevention efforts to slow the progression of additional MCCs and ADL limitations may improve HRQOL among older adults.

For our study, new ADLs 2 years later were independent of baseline ADLs and thus likely represent a real deterioration in health over time. Additionally, the strong relationship between MCCs and increased ADL limitations suggests that declines in function resulted from these MCCs (22).

Besides significant indirect effects on HRQOL through ADL limitations, MCCs also directly affected changes in HRQOL during a 2-year period. The number of baseline MCCs and the changes in MCCs were both strongly associated with changes in physical and mental HRQOL. Therefore, although MCCs predict changes in ADL limitations, they also directly affect HRQOL, independent of older adults’ functional status. This study suggests that preventing MCCs will decrease the likelihood that older adults will develop additional functional limitations. Furthermore, the associations between physical activity and better HRQOL and between smoking and worse HRQOL are stronger for individuals with functional limitations than those without functional limitations (23). From a public health and clinical treatment perspective, encouraging physical activity and smoking cessation among older adults, particularly those with functional limitations, may be valuable in both reducing MCCs and their associated functional limitations and improving HRQOL.

Community-based strategies and policies such as those that increase physical activity and reduce risk of falls can successfully enhance functioning and quality of life among older adults (24). Furthermore, entities such as CDC’s Healthy Aging Research Network (www.prc-han.org), in partnership with public health and the aging services networks, have been translating evidence-based programs and policies into practice in communities across the country (25,26). Additional studies are needed to examine these relationships within transitions of care across providers for people at high risk, including the associated costs, particularly for those living with MCCs. These results also highlight the need to increase interactions between the public health research community and organizations such as the Healthy Aging Research Network to assist in translating and disseminating aging research methods and findings for health promotion and aging initiatives.

This study has several limitations and strengths. This study included only people enrolled in MA Plans; therefore, our sample likely included older adults from higher socioeconomic status backgrounds who are healthier than those not enrolled in these optional plans (27). Additionally, because of the longitudinal nature of the study, people who died before follow-up were not included. Therefore, these findings may not generalize to other older adults without this type of additional health care coverage, those in the worst health, or older adults of lower socioeconomic status. Furthermore, because this sample was limited to those aged 65 or older, these findings may not apply to younger adults.

Despite these limitations, this study had numerous strengths. First, this study analyzed longitudinal data to monitor changes in the health status of a very large sample of community-dwelling older adults. This study also used established measures of MCCs, ADLs, and HRQOL and simultaneously estimated the direct and indirect associations among changes in MCCs, ADLs, and HRQOL. Finally, this study built on prior literature that had relied primarily on cross-sectional data (eg, Johnson and Wolinsky [10]) and on data that did not assess the impact of functional limitations on HRQOL (eg, Lawrence and Jette [12]). Future studies may benefit from monitoring changes in MCCs and functional limitations over longer periods of time and from including younger adults and those in poorer health.

These findings support the need for practitioners to monitor and strive to reduce the number of MCCs experienced by their patients to facilitate optimal function and HRQOL among older adults. Practitioners should recognize the need for better care coordination, particularly for those with MCCs to ensure that conditions do not go untreated, to prevent the acquisition of new conditions, and to limit the burden of disease (6,28). This need for better care coordination may require the removal of barriers that often contribute to “siloed” care for chronic disease, including revisions to the fee-for-service reimbursement structure and the need to reward providers who communicate across sites of care (6,28). Reduction of MCCs and prevention of new MCCs may slow the onset and the progression of functional limitations, thereby further aiding in the maintenance of better HRQOL. Future studies would benefit from longer follow-up periods, the study of younger people and those in poorer health, and the assessment of whether specific combinations of MCCs have stronger associations with declines in functional limitations and HRQOL over time.
